# Non-Smooth Control Barrier Navigation Functions for STL Motion Planning

**DOI:** 10.3389/frobt.2022.782783

**Published:** 2022-04-13

**Authors:** Ashkan Zehfroosh, Herbert G. Tanner

**Affiliations:** Department of Mechanical Engineering, University of Delaware, Newark, DE, United States

**Keywords:** signal temporal logic, robot motion planning, control barrier function, navigation function, autonomous systems

## Abstract

This paper reports on a new approach to Signal Temporal Logic (STL) control synthesis, that 1) utilizes a navigation function as the basis to construct a Control Barrier Function (CBF), and 2) composes navigation function-based barrier functions using nonsmooth mappings to encode Boolean operations between the predicates that those barrier functions encode. Because of these two key features, the reported approach 1) covers a larger fragment of STL compared to existing approaches, 2) alleviates the computational cost associated with evaluation of the control law for the system in existing STL control barrier function methodologies, and 3) simultaneously relaxes some of the conservativeness of smooth combinations of barrier functions as a means of implementing Boolean operators. The paper demonstrates the efficacy of this new approach with three simulation case studies, one aiming at illustrating how complex STL motion planning specification can be realized, the second highlights the less-conservativeness of the approach in comparison to the existing methods, and another that shows how this technology can be brought to bear to push the envelope in the context of human-robot social interaction.

## 1 Introduction

As soon as an infant starts moving she begins to perceive the world in fundamentally different ways (Higgins et al., 1996). This is because children’s spatial knowledge (how to move in space, avoid obstacles, estimate distances, find hidden objects, and decide which surfaces can bear weight) depends on their ability to locomote (Campos et al., 2000; Clearfield, 2004). Infants make mental models by computing spatial relations between their own body and other moving objects (Goksun et al., 2010). Children with motor disabilities are therefore at a developmental disadvantage. Access to power mobility is typically available after the age of 4 or 5 and without early intervention, most of these children will have permanently lost the constant and daily richness of the early years. Infant motor delays can thus have lifelong social and economical consequences, not only for the families, but also for the society as a whole.

In robotic-assisted enriched pediatric rehabilitation environments for infants with motor disabilities, our group has been utilizing robots as a means of encouraging infants to stay engaged in active game-play, in which they explore their environment as well as the capabilities of their own bodies (Kokkoni et al., 2020). In the context of this type of human-robot interaction, preliminary work (Zehfroosh et al., 2017; Zehfroosh and Tanner, 2019) has offered some evidence that in this context, appropriate and effective robot reactions to children behavior are modeled more effectively by temporal (as opposed to static, propositional) logic. In contrast to other branches of temporal logic that like Linear Temporal Logic (LTL), STL is interpreted over continuous-time signals (Maler and Nickovic, 2004) while still capturing timing constraints associated with complex tasks. This feature makes STL an even more appropriate choice for defining robot tasks within time-limited pediatric motor rehabilitation sessions.

Motion planning with STL specifications is known to be hard and usually leads to computationally demanding solutions (Lindemann and Dimarogonas, 2018). The starting thread of work on STL motion planning relies on a computationally demanding mixed-integer linear programming process (Raman et al., 2014; Sadraddini and Belta, 2015; Liu et al., 2017). The computational complexity of these methods makes real-time implementation particularly challenging, especially in the presence of dynamic obstacles (Gundana and Kress-Gazit, 2021). Not surprisingly, Jones et al. (2019) pre-compute the control before execution to overcome real-time implementation issues, at the cost of sensitivity to run-time disturbances.

Lindemann and Dimarogonas (2018) reduced the computational burden of STL motion-planning through a CBF based methodology (Ames et al., 2019). Their method can account for a fragment of STL that includes conjunctions in the predicates or the temporal operators. The control design involves the solution of a Quadratic Programming (QP) problem at each motion planning step. Subsequent extensions of this CBF-based STL motion planning method were made along the directions of multi-agent systems with conflicting local specifications (Lindemann and Dimarogonas, 2019a), and dynamically coupled multi-agent systems (Lindemann and Dimarogonas, 2020).

In this existing STL control design framework, combining predicates and encoding them by (smooth) CBFs introduces a degree of conservatism. This is because of the way the CBF that incorporates the different predicates is constructed, as an exponential summation of component CBFs. Interestingly, one can compose CBF using nonsmooth operators (Glotfelter et al., 2017). Naturally, this comes at the cost of introducing a level of analytical complexity that makes analysis and control design more challenging. Nonsmooth CBFs formulations now exist for time-varying problem instantiations, where different predicates are combined in the form of pointwise minima and maxima of sets of component CBFs (Glotfelter et al., 2019).

Nonsmooth CBFs can relax the conservativeness of predicate composition, but currently share the same method for computing control laws as do smooth CBFs for STL motion planning synthesis (Glotfelter et al., 2017), i.e., solving a QP problem at each iteration of the control loop. Currently, the only STL motion planning method that circumvents the computational burden of solving a optimization problem at every iteration is the funnel-based procedure that provides continuous-time control laws (Lindemann et al., 2017; Lindemann and Dimarogonas, 2019b, 2021). The downside of the aforementioned approach is that it covers a much smaller fragment of STL

This paper is the first to utilize nonsmooth CBFs in STL motion planning and control, circumventing, at least in part, computational control design problems by utilizing navigation functions (Rimon and Koditschek, 1992). Within this new framework, the reported methodology provides directly closed-form control laws that result in a feasible and safe robot paths. This new method 1) considerably alleviates the computation burden related to the solution of a QP optimization problem for attaining control input at every time step, 2) covers a larger class of STL (those that include disjunctions) compared to the existing barrier-function based methods and 3) relaxes some of the conservatism associated with existing CBF composition operations by under-approximating of the minimum operator for the sake of smoothness. What is more, the proposed method allows the incorporation of both attractive and repulsive for the system regions within the same analytical expression, thus reducing the size of the STL specification for a desired task. For clarity of exposition, the present paper describes the methodology as it applies to *sphere world* environments, with pathways to extensions to star world (Li and Tanner, 2018), as well as time-varying robot workspaces (Sun and Tanner, 2015; Chen et al., 2020) readily available.

The rest of this paper is organized as follows: It starts by some technical preliminaries on both STL semantics and non-smooth CBF. Section 3 introduces the problem of interest in the paper by specifying the fragment of STL specifications that motion planning need to be done for. Control barrier navigation functions are presented in Section 4 as a solution to the problem, where both their construction steps as well as the control input computation procedure are elaborated. Finally, Section 5 offers simulation results that illustrate the performance of the technical approach.

## 2 Technical Preliminaries

This section introduces necessary mathematical background needed for the subsequent technical discussion. The section briefly reviews STL non-smooth CBFs, and some known results that will be utilized in following sections. Before these concepts are introduced, let us present the technical terminology for the systems at hand. To that end, let $x\in R^{n}$ denote the state of a dynamical system with input $u\in U\subset R^{m}$ , and let its dynamics be in the form

$$\dot{x}=f\left(x\right)+g\left(x\right)u\;,$$ (1)

and assume that functions $f:R^{n}\to R^{n}$ and $g:R^{n}\to R^{n\times m}$ are locally Lipschitz continuous.

When every solution to (1) which starts in a set stays there, then this set is said to be *forward invariant* relative to (1). Specifically, we say that a set C(t) is forward invariant with respect to (1) if $x\left(t_{0}\right)\in C\left(t\right)\;\Rightarrow \;x\left(t\right)\in C\left(t\right)$ , $\forall \;t\in \left[t_{0},t_{1}\right]\subset R_{+}$ .

**Table:** 

(*x*, *t*)⊧*μ*	iff	*h* (*x*, *t*) ≤ 0
(*x*, *t*)⊧*¬ φ*	iff	$\neg \;\left(\left(x,t\right)⊧\varphi \right)$
(*x*, *t*)⊧*φ*_1_ ∧ *φ*_2_	iff	(*x*, *t*)⊧*φ*_1_ and (*x*, *t*)⊧*φ*_2_
(*x*, *t*)⊧*φ*_1_ ∨ *φ*_2_	iff	(*x*, *t*)⊧*φ*_1_ or (*x*, *t*)⊧*φ*_2_
(*x*, *t*)⊧♢_[*a*,*b*]_ *φ*	iff	*∃ t*_1_ ∈ [*t* + *a*, *t* + *b*] s.t. (*x*, *t*_1_)⊧*φ*
(*x*, *t*)⊧□_[*a*,*b*]_ *φ*	iff	*∀ t*_1_ ∈ [*t* + *a*, *t* + *b*], (*x*, *t*_1_)⊧*φ*
(*x*, *t*)⊧*φ*_1_ *U*_[*a*,*b*]_ *φ*_2_	iff	*∃ t*_2_ ∈ [*t* + *a*, *t* + *b*] s.t. (*x*, *t*_2_)⊧*φ*_2_ and *∀ t*_1_ ∈ [*t* + *a*, *t*_2_], (*x*, *t*_1_)⊧*φ*_1_

There can be cases where the (closed loop) right hand side of (1) needs to be discontinuous in *x*—and therefore cannot be locally Lipschitz. In these cases, both the expression of dynamics, as well as the trajectories of the system, need to be understood in a more general sense. One option for expressing dynamics with *input discontinuities* is by utilizing *differential inclusions* expressed in the form of the Filippov set-valued map, where one would write *F* [⋅]

$$\dot{x}\in F\left[f+g\;u\right]\left(x,t\right)=\bar{co}\left\{\underset{i\to \infty }{\lim}f\left(x_{i}\right)+g\left(x_{i}\right)u\left(x_{i},t_{i}\right),\;S∌\left(x_{i},t_{i}\right)\to \left(x,t\right)\right\}\;,$$ (2)

using $\bar{co}$ to express the convex closure of a set, and *S* to denote a Lebesgue zero-measure set where *f* + *g u* is discontinuous.

The solutions of (2) can be understood in a number of ways, and are generally not unique. One notion of solution is in the sense of Carathéodory; if the Filippov set is nonempty, compact, and convex, and the set-valued map *x*↦*F* (*t*, *x*) is upper-semicontinuous while the set-valued map *t*↦*F* (*t*, *x*) is measurable, then it is known that such Carathéodory solutions for (2)exist (Bacciotti and Rosier, 2005). A Carathéodory solution to (2) on an interval $\left[t_{0},t_{1}\right]\subset R_{+}$ is an absolutely continuous map *x*(*t*) such that $\dot{x}\left(t\right)$ satisfies (2) for almost all *t* ∈ [*t*_0_, *t*_1_]. In what follows, solutions to (2) are understood in this way. Similarly, we say that a set C(t) is forward invariant with respect to (2) if every Carathéodory solution of (2) starting from $x\left(t_{0}\right)\in C\left(t_{0}\right)$ satisfies x(t)∈C(t) for almost all *t* ∈ [*t*_0_, *t*_1_].

### 2.1 Signal Temporal Logic

Signal temporal logic (STL) is a temporal logic formalism that involves logical *predicates*, denoted *μ*, whose truth values are evaluated over continuous signals. In this particular case, the continuous signals are the system’s state trajectories at time *t*, namely *x*(*t*). The predicates assume their logical valuates based on a (continuous) *predicate function* $h:R^{n}\times R_{+}\to R$ as in

$$\mu :=\left\{\begin{array}{cc} True\; & \text{if }h\left(x,t\right)\leq 0\\ False\; & \text{if }h\left(x,t\right)>0\;. \end{array}\right.$$ (3)

Based on such predicates, an STL *formula* *φ* can be recursively defined as

$$\varphi ::=True\;|\;\mu \;|\;\neg \;\varphi \;|\;\varphi_{1}\wedge \varphi_{2}\;|\;\varphi_{1}\vee \varphi_{2}\;|\;{♢}_{\left[a,b\right]}\;\varphi \;|\;{□}_{\left[a,b\right]}\;\varphi \;|\;\varphi_{1}\;U_{\left[a,b\right]}\;\varphi_{2}\;,$$

where $a,b\in R_{+}$ with *a* ≤ *b* are timing bounds, *¬* represents negation, ∧ expresses conjunction, ∨ denotes disjunction, ♢ stands for *eventually*, □ stands for *always* and *U* denotes the *until* temporal operator (Maler and Nickovic, 2004).

If a solution $x:R_{+}\to R^{n}$ of (1) satisfies an STL specification *φ* at time *t*, then we write (*x*, *t*)⊧*φ*. The STL semantics are recursively given by the above (top of this page) rules.

### 2.2 Nonsmooth Control Barrier Functions

A CBF enables controller synthesis for dynamic systems in a way that ensures that if the system starts inside a set, it will never leave that set, rendering the set forward invariant with respect to the dynamics of system. A CBF can characterize the set of allowable control inputs that guarantee forward invariance of certain regions for a dynamical system at hand. The required control input is picked from a set defined in terms of the CBF for example by solving an optimization problem in a sampled-data fashion (Ames et al., 2016).

Nonsmooth CBFs allow more flexibility in the encoding of state constraints and specifications compared to their smooth counterparts. The utilization of such functions typically leads to consideration of the dynamics of the system in the form (2), primarily due to the discontinuities introduced by the control law *u* when it depends on the gradient of a nonsmooth CBF In fact, since the latter are nonsmooth, their gradient cannot be defined everywhere in the usual way. At points of nondifferentiability, one can understand their gradient as a set, rather than a singleton vector, and express it using the concept of the *generalized gradient*, which in finite dimensional spaces enjoys the following concrete characterization as Theorem 1

THEOREM 1 [(Clarke, 1990, Theorem 2.5.1)]. *Consider a locally Lipschitz function* $b:R^{n}\times \left[t_{0},t_{1}\right]\to R$ *. Let* *S* *be any set of Lebesgue measure zero in* $R^{n+1}$ *and* $\Omega_{b}$ *denote the zero-measure set where* b *is non-differentiable. Then, with* $\bar{co}\left\{A\right\}$ *denoting the closure of the convex hull of set* *A* *, the generalized gradient* ∂b(x,t) *of* b *at point* (*x*, *t*) *can be written in terms of the limits of sequences* (*x*_*i*_, *t*_*i*_) → (*x*, *t*) *as follows*

$$\partial b\left(x,t\right)=\bar{co}\left\{\underset{i\to \infty }{\lim}{\left(\begin{array}{cccc} \frac{\partial b}{\partial x_{1}} & \ldots & \frac{\partial b}{\partial x_{n}} & \frac{\partial b}{\partial t} \end{array}\right)}^{⊺}:S\cup \Omega_{b}∌\left(x_{i},t_{i}\right)\to \left(x,t\right)\right\}.$$ (4)

Examples of nonsmooth functions include the point-wise minimum or maximum of a finite collection of locally Lipschitz functions. Indeed, these specific nonsmooth functions are of particular interest in the context of STL synthesis because they can capture the conjunction and disjunction of a number of predicates, when each of the latter is expressed by its own component CBF

In particular, suppose $b\left(x,t\right)=\max_{i\in \left\{1,\ldots ,k\right\}}\left\{b_{i}\left(x,t\right)\right\}$ , where each $b_{i}$ is Lipschitz near (*x*, *t*) (i.e., locally Lipschitz around (*x*, *t*)). Then b is Lipschitz near (*x*, *t*), and if one denotes *I* (*x*, *t*) the set of indices *i* for which $b\left(x,t\right)=b_{i}\left(x,t\right)$ then (Clarke, 1990, Proposition 2.3.12):

$$\partial \underset{i\in \left\{1,\ldots ,k\right\}}{\max}\left\{b_{i}\left(x,t\right)\right\}\subseteq co\left\{\partial b_{i}\left(x,t\right)\;|\;i\in I\left(x,t\right)\right\}\;,$$ (5)

with equality holding if $b_{i}$ is regular at *x* for all *i* ∈ *I* (*x*, *t*), in which case b is also regular. Similarly, if $b\left(x,t\right)=\min_{i\in \left\{1,\ldots ,k\right\}}\left\{b_{i}\left(x,t\right)\right\}$ and each $b_{i}$ is Lipschitz near (*x*, *t*), then again, b is Lipschitz near (*x*, *t*), and

$$\partial \underset{i\in \left\{1,\ldots ,k\right\}}{\min}\left\{b_{i}\left(x,t\right)\right\}\subseteq co\left\{\partial b_{i}\left(x,t\right)\;|\;i\in I\left(x,t\right)\right\}\;,$$ (6)

with equality holding now if $-b_{i}$ is regular at *x* for all *i* ∈ *I* (*x*, *t*), in which case −b is regular too.

Note that although the pairwise maximum of a finite set of continuously differentiable functions is regular, the pairwise minimum function may not be. Nonetheless, if all $b_{i}$ are differentiable at *x*, at least the generalized gradient can be computed in both cases in an expedient manner.

When either the dynamics of a system or the gradient of a function is set valued, the Lie (directional) derivative of the function along the solutions of the system will also be set-valued. Strong (Bacciotti and Ceragioli, 1999) and weak (Bacciotti and Ceragioli, 2006; Glotfelter et al., 2017) versions of set-valued Lie derivatives have been introduced depending on the regularity of the function being differentiated. In this paper we utilize the weak version, at the expense of more relaxed convergence conditions, because we need to consider generalized gradients of functions that may not be regular.

With ⟨⋅, ⋅⟩ denoting the inner product of two vectors, the weak set-valued Lie derivative of a scalar locally Lipschitz function b is now defined as

$$L_{F}^{W}b\left(x,t\right)=\left\{\eta \in R|\exists \;\nu \in F\left[f+gu\right],\exists \;\xi \in \partial b\left(x,t\right)\;\text{ s.t. }\;\langle \xi ,\left[\begin{array}{c} \nu \\ 1 \end{array}\right]\rangle =\eta \right\}\;.$$ (7)

The following lemma (Lemma 1) links time derivative of function b along the solutions of (2) to the weak set-valued Lie derivative of b(x,t) :

LEMMA 1 [cf. (Glotfelter et al., 2019)]. *Consider a Carathéodory solution* $x:\left[0,t^{\prime }\right]\to D\subset R^{n}$ *to the differential inclusion* (2)*, and let* $b:R^{n}\times \left[0,t^{\prime }\right]\to R$ *be a locally Lipschitz function. Then,*

$$\dot{b}\left(x,t\right)\in L_{F}^{W}b\left(x,t\right)\;\text{a.e.}$$ (8)

For a locally Lipschitz scalar function $b:D\times \left[0,t^{\prime }\right]\to R$ with $D\subset R^{n}$ , consider the associated set

$$C=\left\{\left(x,t\right)\in R^{n}\times R_{+}|x\in D,\;b\left(x,t\right)\geq 0\right\}\;.$$

Now the notion of forward invariance can be linked to the concept of CBF through the following definition (Definitions 1,2):

DEFINITION 1 [cf. (Glotfelter et al., 2017, Def. 4) and (Lindemann and Dimarogonas, 2018, Def. 3)]. *A continuous scalar function* $b:D\times \left[0,t^{\prime }\right]\to R$ *where* $D\subset R^{n}$ *is a candidate nonsmooth* *CBF* *if for all* (x,0)∈C *, there exists a Carathéodory solution to* (2) *such that* (x,t)∈C *for all* *t* ∈ [0, *t*′]*.*

DEFINITION 2 [cf. (Glotfelter et al., 2017, Def. 3) and (Lindemann and Dimarogonas, 2018, Def. 2)]. *A continuous candidate nonsmooth* *CBF* $b:D\times \left[0,t^{\prime }\right]\to R$ *where* $D\subset R^{n}$ *is a valid nonsmooth* *CBF* *for* (2)*, if for any* (x,0)∈C *there exists a class-* KL *function* $\beta :R_{+}\times R_{+}\to R_{+}$ *such that*

$$b\left(x\left(t\right),t\right)\geq \beta \left(b\left(x\left(0\right),0\right),t\right)\;\forall t\in \left[0,t^{\prime }\right]$$

*for all Carathéodory solutions of* (2) *starting from* *x* (0)*.*

The following theorem gives a useful equivalent condition for a valid CBF

THEOREM 2 [cf. (Glotfelter et al., 2017, Thm. 2)]. *Let* $D\subset R^{n}$ *be an open and connected set, and* $b:D\times \left[0,t^{\prime }\right]\to R$ *a locally Lipschitz candidate nonsmooth* *CBF* *If there exists a locally Lipschitz extended class-* K *function* α:R→R *such that*

$$\min L_{F}^{W}b\left(x\left(t\right),t\right)\left.\right)\geq -\alpha \left(b\left(x,t\right)\right)\;,\;\forall \left(x,t\right)\in D\times \left[0,t^{\prime }\right]\;,$$ (9)

*then* b *is a valid non-smooth* *CBF* *for* (2)*.*

In the special case where the differential inclusion (2) reduces to a singleton and *g*(*x*)*g*(*x*)^⊺^ is positive definite (so that a simple feedback transformation can bring (1) to the form $\dot{x}=u$ ), a straightforward application of Theorem 2 leads to Corollary 1

COROLLARY 1 *A candidate non-smooth* *CBF* b(x,t) *is a valid non-smooth* *CBF* *for* (1) *if there exists a locally Lipschitz extended class-* K *function* α:R→R *such that*

$$\underset{u\in U}{\sup}\dot{b}\left(x\left(t\right),t\right)\geq -\alpha \left(b\left(x,t\right)\right)\;.$$

In other words, for a valid non-smooth CBF there is always a control input *u* to make the set C(t) forward invariant.

## 3 Problem Statement

This paper considers the following fragment of STL

$$\begin{array}{c} \psi ::=True\;|\;\mu \;|\;\psi_{1}\wedge \psi_{2}\;|\;\psi_{1}\vee \psi_{2} \end{array}$$ (10a)

$$\begin{array}{c} \varphi ::={♢}_{\left[a,b\right]}\;\psi \;|\;{□}_{\left[a,b\right]}\;\psi \;|\;\psi_{1}\;U_{\left[a,b\right]}\;\psi_{2}\;|\;\varphi_{1}\wedge \varphi_{2}\;|\;\varphi_{1}\vee \varphi_{2}\;, \end{array}$$ (10b)

where formulae *ψ*_1_ and *ψ*_2_ are of the type defined in (10a), and formulae *φ*_1_, *φ*_2_ are of the type defined in (10b). This is a larger class of STL compared to Lindemann and Dimarogonas (2018) as it allows for disjunctions in the predicates or the temporal operators.

We make similar assumptions (Assumptions 1,2) on the trajectories and the nature of the term *g*(*x*) in (1):

ASSUMPTION 1 (Lindemann and Dimarogonas (2018)). *For an* *STL* *formula* *φ* *as defined by* (10b)*, there exists a constant* *C* ≥ 0 *such that* (*x*, 0)⊧*φ* ⇒ ‖*x*(*t*)‖ ≤ *C ∀ t* ≥ 0*.*

In other words, satisfaction of formula *φ* guarantees a bounded trajectory.

ASSUMPTION 2 [Lindemann and Dimarogonas (2018)]. *The vector function* *g*(*x*) *in* (1) *is such that* *g*(*x*)*g*(*x*)^⊺^ *is positive definite for all* x∈D *.*

Now, the problem under consideration of this paper can be stated as Problem 1

PROBLEM 1 *Find an input control law* *u*(*x*, *t*) *that guarantees the solution(s)* $x:R_{+}\to R^{n}$ *of* (2) *starting from* *x*_0_ = *x*(0) *be such that* (*x*, 0)⊧*φ* *.*

## 4 Technical Approach

This section introduces a time-varying and nonsmooth CBF that is constructed following the original principles of navigation functions set forth by Rimon and Koditschek (1992). The reported construction leverages the navigation function properties of the CBF to yield a direct method for obtaining the control law *u* in (2) that is guaranteed to satisfy the desired STL specification.

### 4.1 Navigation Functions as Control Barrier Functions

This section borrows primarily from Sun and Tanner (2015), based on the foundation of *sphere world* navigation functions of Rimon and Koditschek (1992), to construct a time-varying CBF with navigation function properties. While Sun and Tanner (2015) allow for time-varying destination configurations, and Chen et al. (2020) consider time-varying obstacle locations, here the construction of the navigation function component of the CBF is itself time-invariant, just as in the original methodology (Rimon and Koditschek, 1992), although time-varying extensions appear plausible (Sun and Tanner, 2015; Chen et al., 2020).

An STL specification consists of logical predicates *μ* as in (3) that can be interpreted as different regions of interest in the state space of the dynamical system at hand, which need to be visited or avoided at particular time periods. Working in a sphere world, all regions of interest (those that a robot needs to approach or those it needs to avoid) are assumed to have spherical shapes. This assumption does not limit the generality of the approach since both Rimon and Koditschek (1992) for the time-invariant case, as well as Li and Tanner (2018) for the case of time-varying destinations, show that diffeomorphic transformations can extend navigation function properties from sphere to (forests of) star worlds.

The key feature of the construction of Sun and Tanner (2015) that is adopted here is the non-point destination. Specifically, instead of the target of navigation being the convergence to a single point, Sun and Tanner (2015) allow for a destination *manifold* in the shape of a spherical shell, which is the zero level set of the function

$$h_{i}\left(x\left(t\right)\right)=\|x\left(t\right)-x_{c_{i}}{\|}^{2}-r_{i}^{2}\;,$$ (11)

which serves as the predicate function encoding logical predicate *μ*_*i*_. In the above, one distinguishes the predicate function’s center $x_{c_{i}}$ and its radius *r*_*i*_. Consistent with STL semantics (3), *μ*_*i*_ is true when *h*_*i*_(*x*) ≤ 0 and false otherwise. Regions of the robot’s workspace that always need to be avoided can be encoded as (static) obstacles and incorporated all together in a specific functional representation inspired by Rimon and Koditschek (1992). Specifically, assuming that the implicit representation of each one of those isolated (obstacle) regions is defined as a function

$$\zeta_{j}\left(x\right)=\|x\left(t\right)-x_{{\text{ob}}_{j}}{\|}^{2}-r_{{\text{ob}}_{j}}^{2}\;j=1,\ldots ,M\;,$$

where $x_{{\text{ob}}_{j}}$ and $r_{{\text{ob}}_{j}}$ denote the center and radius of each undesirable spherical (obstacle) region. Our understanding is that obstacles are being avoided as long as *ζ*_*i*_(*x*) > 0. Similarly, the boundary of the workspace itself is captured by the function

$$\zeta_{0}\left(x\right)=-\|x\left(t\right)-x_{\text{ws}}{\|}^{2}+r_{\text{ws}}^{2}\;,$$

where *x*_ws_ and *r*_ws_ stand for the center and radius of the workspace, respectively. Given these constructs and the fact that all obstacles are assumed to be disjoint, the combined obstacle representation can take the form

$$\zeta \left(x\right)=\overset{M}{\underset{j=0}{\prod }}\zeta_{j}\left(x\right)\;,$$

and with that, a navigation function *ϕ*_*i*_(*x*) can be explicitly constructed for predicate *μ*_*i*_ as

$$\phi_{i}\left(x\right)=\frac{h_{i}\left(x\right)}{{\left[h_{i}{\left(x\right)}^{\kappa }+\zeta \left(x\right)\right]}^{1/\kappa }}\;,$$ (12)

with *κ* = 2*n* for n∈N in the role of a positive tuning constant which be set sufficiently high to guarantee navigation function properties for (12). Note that for all *x* that do not satisfy *μ*_*i*_, it is 0 < *ϕ*_*i*_(*x*) ≤ 1, and ∇*ϕ*(*x*) is non-zero almost everywhere (with the exception of a finite number *M* of isolated critical points).

The following examples illustrate how (12) can be used to construct a time-varying nonsmooth CBF b(x,t) that can encode a *combination* of predicates *μ*_*i*_.

EXAMPLE 1 *Consider the* *STL* *formula* *φ* = ♢_[*a*,*b*]_ *μ*_1_ *. If* *ϕ*_1_(*x*) *is defined as in* (12) *with* *h*_*i*_(*x*) *being the predicate function for* *μ*_1_ *, then a* *CBF* *that captures* *φ* *as a specification can be constructed in the form* $b\left(x,t\right)=1-\phi_{1}\left(x\right)-c_{1}\left(t\right)$ *, where* $c_{1}:R_{+}\to \left[0,1\right]$ *is a nondecreasing function satisfying* *c*_1_(0) = 0 *and* *c*_1_(*t*′) = 1 *for some* *t*′ ∈ [*a*, *b*]*. Then for* *c*_1_(*t*′) = 1*,* $b\left(x\left(t^{\prime }\right),t^{\prime }\right)\geq 0$ *when* $\phi_{1}\left(x\left(t^{\prime }\right)\right)\leq 0$ *, which in turn happens only when* $h_{1}\left(x\left(t^{\prime }\right)\right)\leq 0$ *, implying that* *μ*_1_ *is true.*

EXAMPLE 2 *Consider the* *STL* *formula* *φ* = *φ*_1_ ∧ *φ*_2_ *where* $\varphi_{1}={♢}_{\left[a_{1},b_{1}\right]}\mu_{1}$ *and* $\varphi_{2}={□}_{\left[a_{2},b_{2}\right]}\mu_{2}$ *. Start off by constructing a separate* *CBF* *for each of the two component formulae:* $b_{1}\left(x,t\right)=1-\phi_{1}\left(x\right)-c_{1}\left(t\right)$ *for* *φ*_1_ *, exactly as in* *Example* 1 *, and* $b_{2}\left(x,t\right)=1-\phi_{2}\left(x\right)-c_{2}\left(t\right)$ *for* *φ*_2_ *, where* $c_{2}\left(t\right):R_{+}\to \left[0,1\right]$ *is a nondecreasing function with* *c*_2_(0) = 0 *and* *c*_2_(*t*′) = 1 *for all* *t*′ ∈ [*a*_2_, *b*_2_]*.* ^1^ *The* *CBF* *that expresses* *φ* *can now be formulated as a pointwise minimum of* $b_{1}$ *and* $b_{2}$ *, i.e.,* $b\left(x,t\right)=\min\left\{b_{1},b_{2}\right\}$ *.*

EXAMPLE 3 *Consider the* *STL* *formula* *φ* = *φ*_1_ ∨ *φ*_2_ *where* *φ*_1_ *and* *φ*_2_ *are as in* *Example* 2 *.* *CBF* *s* $b_{1}\left(x,t\right)$ *and* $b_{2}\left(x,t\right)$ *for* *φ*_1_ *and* *φ*_2_ *are constructed exactly as in* *Example* 2 *. However, this time the overall* *CBF* *is formulated as a pointwise maximum of* $b_{1}$ *and* $b_{2}$ *, i.e.,* $b\left(x,t\right)=\max\left\{b_{1},b_{2}\right\}$ *.*

The construction of the CBF based on navigation function (12) provides number of advantages compared to existing CBF-based STL motion planning methods [e.g., (Lindemann and Dimarogonas, 2018)]; First note that since the navigation function can encode unsafe regions (obstacles) through *ζ*(*x*), it obviates the need for the explicit definition of additional logical predicates corresponds to such unsafe regions, thus reducing the size of the STL specification. This reduction in the size of STL is particularly useful if this method is used in conjunction with a reactive STL (event-based STL) motion planning methodology (Gundana and Kress-Gazit, 2021) that includes a prior higher-level automata synthesis step. Another advantage of the nonsmooth formulation is that not only paves the way for covering larger class of STL compared to those considered by Lindemann and Dimarogonas (2018), but also eliminates the conservatism associated with under-approximation of minimum operator for the sake of smoothness (see Section 5.2). Yet another advantage of control barrier navigation functions is related to a reduction of the computational load required for determining control inputs (see Section 4.2).

Based on the idea illustrated in Examples 1, 2 and 3, the following sections present the development of a three-step process to produce CBFs that encode general specifications in the STL fragment (10).

#### 4.1.1 STL Specifications With no Conjunctions and Disjunctions

This section describes how to construct a CBF for an STL specification that does not involve conjunctions and disjuctions of predicates and temporal operators.

If this STL specification in question is of the form ♢_[*a*,*b*]_ *μ*_1_ then the CBF can be constructed as

$$b\left(x,t\right)=1-\phi \left(x\right)-c\left(t\right)\;,$$ (13)

where $c:R_{+}\to \left[0,1\right]$ is a non-decreasing function with *c* (0) = 0 and *c* (*t*′) = 1 for some *t*′ ∈ [*a*, *b*].

If the specification has the form □_[*a*,*b*]_ *μ*, the CBF can have the same general form b(x,t)=1−ϕ(x)−c(t) , only now the non-decreasing function $c:R_{+}\to \left[0,1\right]$ is such that *c* (0) = 0 and *c* (*t*′) = 1 for all *t*′ ∈ [*a*, *b*].

The remaining case refers to specifications of the form *μ*_1_ *U*_[*a*,*b*]_ *μ*_2_, for which the CBF is constructed as

$$b\left(x,t\right)=\min\left\{b_{1},b_{2}\right\}\;,$$ (14)

Where once again $b_{i}\left(x,t\right)=1-\phi_{i}\left(x\right)-c_{i}\left(t\right)$ for *i* ∈ {1, 2} as in (13), with $c_{2}:R_{+}\to \left[0,1\right]$ a non-decreasing function satisfying *c*_2_ (0) = 0 and *c*_2_ (*t*′) = 1 for some *t*′ ∈ [*a*, *b*], while $c_{1}:R_{+}\to \left[0,1\right]$ is a non-decreasing function satisfying *c*_1_ (0) = 0 and *c*_1_ (*t*″) = 1 for all *t*″ ∈ [*a*, *t*′].

#### 4.1.2 STL Specifications With no Conjunctions or Disjunctions Between Temporal Operators

This section refers to STL specifications that may have conjunctions and disjunctions involving predicates but not temporal operators. We assume that the formulae inside a temporal operator has been written in Conjunction Normal Form (CNF), i.e., $\left(\mu_{1}\vee \mu_{2}\vee \ldots \right)\wedge \left(\mu_{1}^{\prime }\vee \mu_{2}^{\prime }\vee \ldots \right)\wedge \ldots$ . Without loss of generality, take two illustrative cases of predicates

$$\psi_{1}=\left(\mu_{1}\vee \mu_{2}\right)\wedge \left(\mu_{3}\right)\;\text{and}\;\psi_{2}=\left(\mu_{4}\vee \mu_{5}\right)\wedge \left(\mu_{6}\right)\;.$$

Then if the specification has the form ♢_[*a*,*b*]_ *ψ*_1_, the CBF can take the form of

$$b\left(x,t\right)=\min\left\{\max\left\{b_{1},b_{2}\right\},b_{3}\right\}\;,$$ (15)

Where each $b_{i}\left(x,t\right)$ is constructed as in (13) for *i* ∈ {1, 2, 3}, and with each $c_{i}:R_{+}\to \left[0,1\right]$ being a non-decreasing function with *c*_*i*_ (0) = 0 and *c*_*i*_ (*t*′) = 1 for some *t*′ ∈ [*a*, *b*].

For specifications of the form □_[*a*,*b*]_ *ψ*_1_, the CBF can be similarly constructed based on (15) with component CBFs as in (13), but this time each $c_{i}:R_{+}$ is a non-decreasing function with *c*_*i*_ (0) = 0 and *c*_*i*_ (*t*′) = 1 for all *t*′ ∈ [*a*, *b*].

Finally, for specifications involving the Until operator and of the form *ψ*_1_ *U*_[*a*,*b*]_ *ψ*_2_, the CBF can be formed as

$$b\left(x,t\right)=\min\left\{\max\left\{b_{1},b_{2}\right\},b_{3},\max\left\{b_{4},b_{5}\right\},b_{6}\right\}\;,$$

where all component CBFs $b_{i}\left(x,t\right)$ are constructed using the basic template (13), but for $i\in \left\{4,5,6\right\}\;c_{i}:R_{+}\to \left[0,1\right]$ are non-decreasing functions satisfying *c*_*i*_ (0) = 0 and *c*_*i*_ (*t*′) = 1 for some *t*′ ∈ [*a*, *b*], while for *j* ∈ {1, 2, 3}, the functions $c_{j}:R_{+}\to \left[0,1\right]$ are also non-decreasing but with *c*_*j*_ (0) = 0 and *c*_*j*_ (*t*″) = 1 for all *t*″ ∈ [*a*, *t*′].

#### 4.1.3 General Case of STL Specifications

Combining the constructions of Sections 4.1.1, 4.1.2, one is now in position to form CBF for more general STL specifications in the fragment defined in (10). Again, we assume that the STL specification is written in CNF with respect to the temporal operators. As an illustrative example, consider the case of $\left({♢}_{\left[a_{1},b_{1}\right]}\psi_{1}\vee {□}_{\left[a_{2},b_{2}\right]}\psi_{2}\right)\wedge \left(\psi_{3}U_{\left[a_{3},b_{3}\right]}\psi_{4}\right)$ . Then the CBF can take the form of (15) where $b_{1}\left(x,t\right)$ , $b_{2}\left(x,t\right)$ and $b_{3}\left(x,t\right)$ are each associated with one of the three temporal operators appearing in the general formula, constructed based on the designs of Section 4.1.1, and then combined according to the rules outlined in Section 4.1.2.

In addition to the ability to cover STL specifications including disjunctions, the construction process outlined in Sections 4.1.1–4.1.3 generally yields less conservative CBFs compared to the method of Lindemann and Dimarogonas (2018), because the latter introduces some conservativeness through its exponential summation to combine the component CBFs (see Section 5.2); the perceived benefit of this latter construction is that it preserves the differentiability properties of the CBF and circumvents the need for nonsmooth analysis. What is more, the computation process described here can be further accelerated by adopting the deletion mechanism of Lindemann and Dimarogonas (2018), whereby a component CBF $b_{i}\left(x,t\right)$ drops from the composite construction b(x,t) whenever time *t* exceeds the upper limit of the time interval of its corresponding temporal operator, say [*a*_*i*_, *b*_*i*_], i.e., *t* > *b*_*i*_. For the Always and Until temporal operators, the associated barrier function is droped whenever its value become negative in the time interval of the operator. The section that follows highlights additional benefit of the nonsmooth construction of CBFs using navigation functions: 1) the navigation function properties of component barrier functions $b_{i}$ , i.e., that the associated (negated) gradient system is guaranteed to converge to the zero level set of the predicate function, is inherited through the composition operations, and 2) the control law that realizes the STL system specification can be derived in a straightforward manner, usually obviating the need for the repeated solution of a QP problem.

### 4.2 Efficient Determination of the Control Input

Section 4.1 primarily illustrated how the use of navigation functions and pointwise minimum functions can allow the construction of CBFs that tightly encode STL specifications in the fragment defined by (10). This section focuses on demonstrating that control design can also be facilitated due to the navigation function properties afforded by the proposed component CBFs.

Without loss of generality, let *p* be the total number of predicates and *q* be the total number of temporal operators appearing in the STL specification. For *j* ∈ {1, … , *q*}, and with the formulae inside the temporal operators written in CNF (Section 4.1.3), then temporal operator indexed *k* will be modelled by a CBF of the form

$$b_{p+j}\left(x,t\right)=\min\{\max\left\{b_{k_{j}}\left(x,t\right),\ldots ,b_{l_{j}}\left(x,t\right)\right\},\ldots ,\max\left\{b_{m_{j}}\left(x,t\right),\ldots ,b_{n_{j}}\left(x,t\right)\right\}\}$$ (16)

For some distinct *k*_*j*_, *l*_*j*_, *m*_*j*_, *n*_*j*_ ∈ {1, … , *p*}. Then the temporal operators of the STL formula can themselves be arranged in CNF (Section 4.1.2), in which case the composite (total) CBF capturing the complete STL specification would take a similar compact form

$$b\left(x,t\right)=\min\{\max\left\{b_{p+k}\left(x,t\right),\ldots ,b_{p+l}\left(x,t\right)\right\},\ldots ,\max\left\{b_{p+m}\left(x,t\right),\ldots ,b_{p+n}\left(x,t\right)\right\}\}$$ (17)

For some other distinct *k*, *l*, *m*, *n* ∈ {1, … , *q*}, with the understanding of each one of the $b_{p+*}$ component CBFs above is of the form (16). Note that all $b_{i}$ with *i* ∈ {1, … , *p*} are continuously differentiable functions, while all $b_{p+j}$ with *j* ∈ {1, … , *q*} are not, but they are still locally Lipschitz functions. In the rest of the paper we refer to those *p* continuously differentiable components of b as *component* CBFs. In view of (16), (17), define the index set of the component CBFs of the form (13) that simultaneously agree with the value of b in (17) as

$$J\left(x,t\right)=\left\{i\in \left\{1,\ldots ,p\right\}\;|\;b\left(x,t\right)=b_{i}\left(x,t\right)\right\}\;.$$

Then the control input *u* that guarantees that b(x,t)≥0 can be computed directly using the gradient of the CBF unless *x* is a point where the latter non-differentiable. At such points, resorting to QP for the determination of the control input *u* may be unavoidable, although there are still cases where such a computationally expensive procedure can be circumvented. The following sections illustrate different options, starting with the straightforward one where b is computed away from points of nondifferentiability.

#### 4.2.1 When the CBF is Differentiable at *x*

When the CBF is differentiable at point *x*, the set *J* is a singleton. Without loss of generality assume that at that time *t*, it is *J* (*x*, *t*) = {1}. Then the control input *u* (*x*, *t*) to guarantee b(x,t)≥0 can be obtained as

$$u\left(x,t\right)=k\;g{\left(x\right)}^{⊺}\;\frac{\partial b_{1}\left(x,t\right)}{\partial x}\;,$$ (18)

Where *k* should be selected such that the following condition, involving an extended class- $K_{\infty }$ function *α*, holds:

$${\frac{\partial b_{1}\left(x,t\right)}{\partial x}}^{⊺}\left(f\left(x\right)+g\left(x\right)u\left(x,t\right)\right)+\frac{\partial b_{1}\left(x,t\right)}{\partial t}\geq -\alpha \left(b\left(x,t\right)\right)\;.$$ (19)

Therefore, *k* can be selected as the maximum between zero and the solution of the equation

$$k\;\left[{\frac{\partial b_{1}\left(x,t\right)}{\partial x}}^{⊺}\;g\left(x\right)g{\left(x\right)}^{⊺}\;\frac{\partial b_{1}\left(x,t\right)}{\partial x}\right]\;=-\alpha \left(b\left(x,t\right)\right)-\frac{\partial b_{1}\left(x,t\right)}{\partial t}-{\frac{\partial b_{1}\left(x,t\right)}{\partial x}}^{⊺}f\left(x\right)\;.$$ (20)

A solution to (20) exists almost everywhere since *g*(*x*)*g*(*x*)^⊺^ is assumed to be positive definite (by Assumption 2) and

$$\frac{\partial b_{j}\left(x,t\right)}{\partial x}=-\nabla_{x}\phi_{j}\left(x\right)\;,$$

and the latter is *guaranteed* to be non-zero almost everywhere away from the level set of *h*_*j*_ (with the exception of a finite number of isolated points). Note that negative solutions for *k* can be safely discarded, since the trivial choice of *k* = 0 would still satisfy (19) and offer an admissible control law with an even smaller (than the negative *k*) norm. Given that in the case considered in this section, (5–8) reduce to singletons, $\dot{x}=f+g\;u$ , and in view of Theorem 2, the choice of *u* (*x*, *t*) given by (18) guarantees that b(x,t)≥0 .

#### 4.2.2 When the CBF is not Differentiable at *x*

At configurations *x* where a CBF is not differentiable, one of the following two cases can occur: precisely two component CBFs agree with the value of b at the same *x*, or more than two components CBFs the value of b simultaneously.

When just two component CBF agree with the value of b , then without loss of generality assume that these are $b_{1}$ and $b_{2}$ in which case *J* (*x*, *t*) = {1, 2}. Then, from (5–8), and Theorem 2 it follows that for all $w_{1},w_{2}\in R_{+}$ such that *w*_1_ + *w*_2_ = 1, the control input *u* (*x*, *t*) needs to satisfy

$$\begin{array}{l} w_{1}{\frac{\partial b_{1}\left(x,t\right)}{\partial x}}^{⊺}\left[f\left(x\right)+g\left(x\right)u\left(x,t\right)\right]+w_{2}{\frac{\partial b_{2}\left(x,t\right)}{\partial x}}^{⊺}\left[f\left(x\right)+g\left(x\right)u\left(x,t\right)\right]\\ +w_{1}\frac{\partial b_{1}\left(x,t\right)}{\partial t}+w_{2}\frac{\partial b_{2}\left(x,t\right)}{\partial t}\geq -\alpha \left(b\left(x,t\right)\right)\;. \end{array}$$ (21)

For (21) to hold, it is sufficient that the following two inequalities are simultaneously satisfied:

$$\left.\begin{array}{cc} & {\frac{\partial b_{1}\left(x,t\right)}{\partial x}}^{⊺}\left[f\left(x\right)+g\left(x\right)u\left(x,t\right)\right]+\frac{\partial b_{1}\left(x,t\right)}{\partial t}\geq -\alpha \left(b\left(x,t\right)\right)\\ & {\frac{\partial b_{2}\left(x,t\right)}{\partial x}}^{⊺}\left[f\left(x\right)+g\left(x\right)u\left(x,t\right)\right]+\frac{\partial b_{2}\left(x,t\right)}{\partial t}\geq -\alpha \left(b\left(x,t\right)\right) \end{array}\right\}\;.$$ (22)

Assume now a control law of the form

$$u\left(x,t\right)=k_{1}\;g{\left(x\right)}^{⊺}\;\frac{\partial b_{1}\left(x,t\right)}{\partial x}+\;k_{2}\;g{\left(x\right)}^{⊺}\;\frac{\partial b_{2}\left(x,t\right)}{\partial x}\;,$$ (23)

Where the control gains *k*_1_ and *k*_2_ are determined as the maximum between zero and the solutions to the following system of algebraic equations:

$$\left[\begin{array}{cc} {\frac{\partial b_{1}}{\partial x}}^{⊺}gg^{⊺}\frac{\partial b_{1}}{\partial x} & {\frac{\partial b_{1}}{\partial x}}^{⊺}gg^{⊺}\frac{\partial b_{2}}{\partial x}\\ {\frac{\partial b_{2}}{\partial x}}^{⊺}gg^{⊺}\frac{\partial b_{1}}{\partial x} & {\frac{\partial b_{2}}{\partial x}}^{⊺}gg^{⊺}\frac{\partial b_{2}}{\partial x} \end{array}\right]\left[\begin{array}{c} k_{1}\\ k_{2} \end{array}\right]=\left[\begin{array}{c} -{\frac{\partial b_{1}}{\partial x}}^{⊺}f-\frac{\partial b_{1}}{\partial t}-\alpha \left(b\right)\\ -{\frac{\partial b_{2}}{\partial x}}^{⊺}f-\frac{\partial b_{2}}{\partial t}-\alpha \left(b\right) \end{array}\right]\;.$$ (24)

The above system of equations always has a unique solution except when $g{\left(x\right)}^{⊺}\;\frac{\partial b_{1}\left(x,t\right)}{\partial x}$ and $g{\left(x\right)}^{⊺}\;\frac{\partial b_{2}\left(x,t\right)}{\partial x}$ are linearly dependent. In the case that $g{\left(x\right)}^{⊺}\;\frac{\partial b_{1}\left(x,t\right)}{\partial x}=\gamma \;g{\left(x\right)}^{⊺}\;\frac{\partial b_{2}\left(x,t\right)}{\partial x}$ with *γ* ≥ 0, one can still substitute *k*_2_ = 0 in (23), plug in (22) (with equality instead of inequality), and solve for *k*_1_ picking the largest possible value for it. In the case^2^ where $g{\left(x\right)}^{⊺}\;\frac{\partial b_{1}\left(x,t\right)}{\partial x}=-\gamma \;g{\left(x\right)}^{⊺}\;\frac{\partial b_{2}\left(x,t\right)}{\partial x}$ , one may ultimately resort to solving the QP [cf. (Glotfelter et al., 2017)]:

$$\begin{array}{cc} & \min\|\hat{u}{\|}^{2}\;such\;that\\ & {\frac{\partial b_{j}\left(x,t\right)}{\partial x}}^{⊺}\left[f\left(x\right)+g\left(x\right)u\right]+\frac{\partial b_{j}\left(x,t\right)}{\partial t}\geq -\alpha \left(b\left(x,t\right)\right)\;,\;\forall j\in J\;. \end{array}$$ (25)

Note that the solution to the above QP coincides with the input derived from (5–8) and Theorem 2.

The case when *J* contains two members does not generalize to instances where more than two component CBFs agree with the value of b simultaneously. A counter example can be constructed for $x\in R^{2}$ and *J* (*x*, *t*) = {1, 2, 3}, in which case the algebraic system of the form (24) (but now with three unknowns *k*_1_, *k*_2_, and *k*_3_) can be shown to either have infinitely many, or no solutions at all. In such rare cases (see Section 5.1), one is still forced to solve (25).

Note that (19), (22) and the optimization constraint in (25) are all equivalent versions of (9) for the cases when *J* = {1}, *J* = {1, 2} and general form of *J*, respectively. According to Theorem 2, then the barrier function will be valid and consequently by Definition 2, it means that if the system starts where b(x,t)≥0 it will always remain in regions that b(x,t)≥0 for all control inputs as (18), (23) or (25).

Note that the existing closed-form solutions to the CBF-based QP only apply to time-invariant safe sets (Ames et al., 2016). This time-invariance is not conducive to STL planning, that often requires that safe sets to change over time. This is why the existing CBF-based STL planning methods (Glotfelter et al., 2017; Lindemann and Dimarogonas, 2018) discretize time and employ QP iteratively in the control loop with an additional assumption [Assumption 3 in Lindemann and Dimarogonas (2018)] on the barrier function to explicitly accommodate safe sets that shrink over time. In contrast, and excluding the singular cases that are dealt by (25), the general process for determining the CBF-based control law outlined above, provides computational benefits because it obviates (25) in all but a very small subset of time steps where the CBF is not differentiable (see Section 5.1).

The following proposition (Proposition 1) states that the control law designs of (18) and (23) are in fact the minimum-norm input that satisfied the required conditions (19) or (22), and thus coincide with the solution of (25). Consequently, when the solutions given by (18) or (23) fail to satisfy an actuation bound, this in fact means that the problem is infeasible in this CBFs framework, given the actuation constraints.

PROPOSITION 1 *The control laws* (18) *and* (23) *give the minimum-norm inputs that satisfy* (19) *and* (22)*, respectively.*

PROOF We prove the claim for (18); the process for (23) is a mirror image. Let u* be the minimum-norm control input that satisfies (19). By contradiction: assume that *u* of (18) is such that ‖*u*‖ > ‖u*‖. Then *both* the following conditions should be satisfied:

$$\left.\begin{array}{cc} & {\frac{\partial b_{1}}{\partial x}}^{⊺}\;g\;u=-\alpha \left(b\right)-\frac{\partial b_{1}}{\partial t}-\frac{\partial b_{1}}{\partial x}f\\ & {\frac{\partial b_{1}}{\partial x}}^{⊺}\;g\;u^{*}\geq -\alpha \left(b\right)-\frac{\partial b_{1}}{\partial t}-\frac{\partial b_{1}}{\partial x}f \end{array}\right\}\;.$$ (26)

The first equation is the consequence of choosing coefficient *k* according to (20). Given now that *u* is by construction aligned to the vector $\frac{\partial b_{1}}{\partial x}g^{⊺}$ , and since ‖*u*‖ > ‖u*‖, the inner product $\left({\frac{\partial b_{1}}{\partial x}}^{⊺}g\right)\;u$ must always be bigger than $\left({\frac{\partial b_{1}}{\partial x}}^{⊺}g\right)\;u^{*}$ , i.e., $\left({\frac{\partial b_{1}}{\partial x}}^{⊺}g\right)\;u>\left({\frac{\partial b_{1}}{\partial x}}^{⊺}g\right)\;u^{*}$ . This contradicts (26).

## 5 Simulation Results

This section is organized in three parts. The objective of the first part, which is Section 5.1, is to demonstrate the capabilities of the reported nonsmooth CBF utilizing a relatively complex STL specification. The second part, i.e., Section 5.2, is to show the less-conservatism of the offered method in comparison to Lindemann and Dimarogonas (2018). The third part, Section 5.3, is to illustrate how the reported technology can be applied in the context of robot-child interaction for pediatric motor rehabilitation purposes within an enriched environment where robots socially interact with infants, thus linking back to the motivating application that opened up Section 1.

### 5.1 Robot Motion Planning With Complex **STL Specifications**

Consider a robot in a 2D spherical workspace of radius 1, initially positioned at a configuration with coordinates *x*_0_ = (0.9,0.2)^⊺^ and with dynamic $\dot{x}=u$ . The workspace contains a static spherical obstacle of radius 0.2236, centered at (0.5,0)^⊺^. The obstacle region is obviously an area that the robot should always avoid.

In addition to avoiding obstacles, the robot has an array of mission objectives associated with different (spherical) regions of interest in its workspace. These mission objectives will naturally be expressed in STL In general, we will denote *μ*_*i*_ the predicate that is associated with the *i*th region of interest. Table 1 collects the topological information of the different regions of interest for the robot.

**TABLE 1:** Geometric characteristics of regions of interest for an STL motion planning task.

Predicate	Center position coordinates	Region radius
*μ*_1_	(−0.1,0)^⊺^	0.3
*μ*_2_	(−0.4,0)^⊺^	0.3
*μ*_3_	(−0.6,0.2)^⊺^	0.3
*μ*_4_	(−0.35,−0.3)^⊺^	0.2
*μ*_5_	(−0.4,−0.6)^⊺^	0.2

The STL task specification that the robot needs to satisfy is given in the following form:

$$\varphi_{1}=\left({□}_{\left[3,7\right]}\left(\mu_{1}\vee \mu_{2}\right)\;\vee \;{♢}_{\left[2,4\right]}\mu_{3}\right)\wedge \left({♢}_{\left[4,5\right]}\left(\mu_{2}\wedge \mu_{3}\right)\right)\wedge \left(\mu_{4}U_{\left[6,10\right]}\mu_{5}\right)\;.$$ (27)

Using the construction process outlined in Sections 4.1.1–4.1.3, the barrier function for (27) is as follows:

$$b=\min\left\{\max\left\{\max\left\{b_{1},b_{2}\right\},b_{3}\right\},\min\left\{b_{2}^{\prime },b_{3}^{\prime }\right\},\min\left\{b_{4},b_{5}\right\}\right\}\;.$$ (28)

Note that while $b_{2}$ (or $b_{3}$ ) and $b_{2}^{\prime }$ (or $b_{3}^{\prime }$ ) are constructed for the same region *μ*_2_ (or *μ*_3_), but due to the *different time intervals* associated with the temporal operators that contain *μ*_2_ (or *μ*_3_), they are in fact different barrier functions, as a result of using a different *c*(*t*) in their construction (see Section 4.1.1).

**FIGURE 1:**
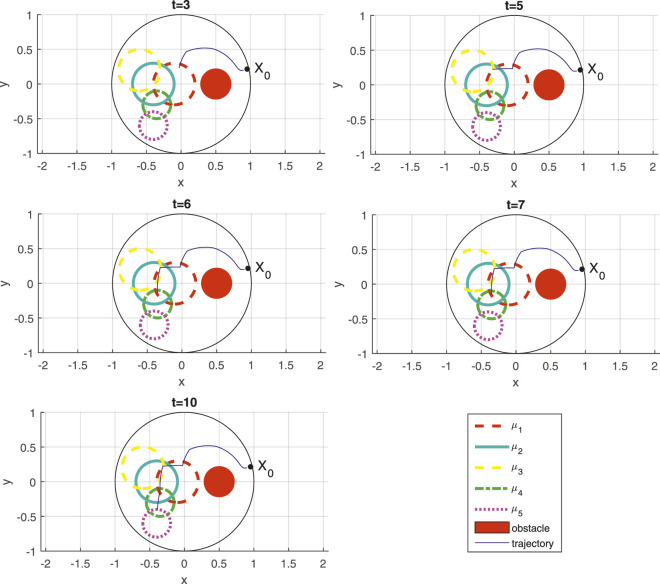
Path of the robot as it is controlled to satisfy STL task specification *φ*_1_ (27) with snapshots at different time instances; the time instant is indicated at the top of each subfigure.

Figure 1 gives successive snapshots of the robot’s path through the workspace, as it is steered by the control law computed based on the process outlined in Section 4.2. The time instances associated with the snapshots showcased correspond to representative moments in relation to the temporal operators appearing in the STL specification *φ*_1_ in (27). First of all, as a result of maximum operator, visiting (*μ*_1_ ∨ *μ*_2_) at *t* = 3 is preferred over visiting *μ*_3_ since the former is much closer to the initial location of the robot. For the same reason, between *μ*_1_ and *μ*_2_, the former is selected to be visited at time *t* = 3. It should continue to remain inside the union of *μ*_1_ and *μ*_2_ (to ensure (*μ*_1_ ∨ *μ*_2_) remains true) from *t* = 3 until *t* = 7, which is verified in the sequence of subsequent snapshots at times *t* = 3, *t* = 5, *t* = 6, and *t* = 7. Meanwhile, however, and sometime in the [4, 5] time interval, the intersection of *μ*_2_ and *μ*_3_ must be visited (to make (*μ*_2_ ∧ *μ*_3_) true), a fact that is evident in the top left snapshot for *t* = 5 where the robot is shown to make a maneuver to the left to reach the intersection of *μ*_2_ and *μ*_3_. Then, the specification *φ*_1_ indicates that in the [6, 10] time interval predicate *μ*_4_ should first be satisfied before predicate *μ*_5_ becomes true. Indeed, the robot is shown at *t* = 6 to have touched the boundary of *μ*_4_; following that, at time instant *t* = 10 the robot is shown to have touched the boundary of *μ*_5_. While all these maneuvers take place, the robot always stays clear of the static obstacle, marked in Figure 1 with the solid red disk.

**FIGURE 2:**
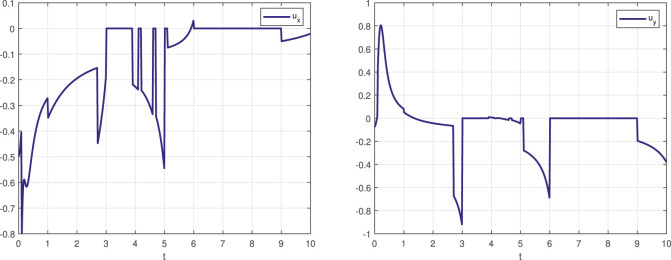
The control input signal (*u*_*x*_ left, *u*_*y*_ right) that realized the STL task specification *φ*_1_ expressed in (27).

Figure 2 presents graphs that show the evolution of the two-dimensional control input *u* (*x*, *t*) that implements the STL task specification (27). As Figure 2 indicates, the control inputs experience discontinuities. Not surprisingly, several jumps occur at time instants coinciding with non-differentiable points of the barrier function.

**FIGURE 3:**
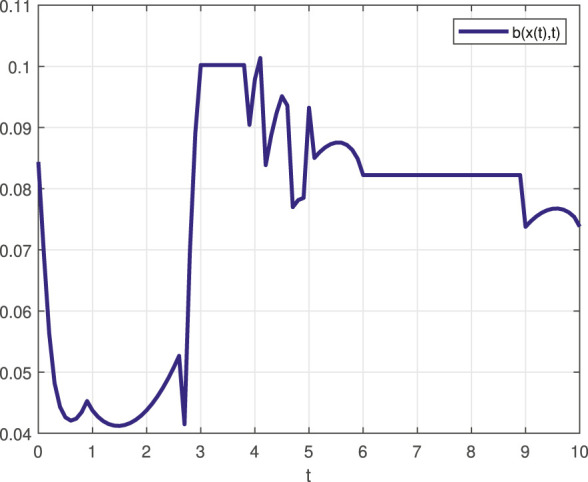
Control barrier function b(x,t) for STL task specification *φ*_1_ (27). The 17 non-differentiable points are corresponds to time steps with *J* (*x*, *t*) containing two members for which the control law (23) still works.

To see better the computational savings of this method compared to approaches that required the repeated solution of the QP program for the determination of the control law, the time interval [0, 10] of the STL task (27) was discretized to 1,000 time steps. Among those, only 17 featured *J* (*x*, *t*) with cardinality two, while there were *zero* instances where |*J* (*x*, *t*)| > 2. Of those 17 time steps, which show as the non-differentiable points of the barrier function depicted in Figure 3, none of them marked a singular case; consequently, (23) applies to them all, and (18) used everywhere else. Therefore, in handling the STL task (27), the reported method *never* resorted to solving a QP problem.

### 5.2 Evidence of Conservatism Relaxation

This section includes an illustrative example that demonstrates how less conservative the presented solution can be [even for the smaller STL class considered by Lindemann and Dimarogonas (2018)] when satisfying STL specifications, specifically in cases where smooth formulations do not permit the satisfaction of these specifications.

Consider a robot in a 2D spherical workspace of radius 3, initially positioned at a configuration with coordinates *x*_0_ = (−2,1)^⊺^. The STL specification of the robot’s mission in this workspace involves visiting two regions of interest whose topological information is presented in Table 2.

**TABLE 2:** Geometric characteristics of regions of interest for an STL motion planning task.

Predicate	Region center position	Radius
*μ*_1_	(0,0)^⊺^	1
*μ*_2_	(1.5,0)^⊺^	1

The STL task specification that the robot needs to satisfy is given in the following form:

$$\varphi_{2}=\left({□}_{\left[1,3\right]}\left(\mu_{1}\right)\right)\wedge \left({□}_{\left[2,4\right]}\left(\mu_{2}\right)\right)\;.$$ (29)

Within a smooth STL composition framework, planning for satisfaction of (29) proceeds as follows (Lindemann and Dimarogonas, 2018): 1) first one defines predicate functions *h*_1_ = 1 − ‖*x*‖ and *h*_2_ = 1 − ‖*x* − (1.5,0)^⊺^‖ for regions *μ*_1_ and *μ*_2_ respectively; 2) then the barrier function for each sub-formula of (29) is constructed as $b_{1}=\gamma_{1}\left(t\right)-\|x\|$ and $b_{2}=\gamma_{2}\left(t\right)-\|x-{\left(1.5,0\right)}^{⊺}\|$ ; 3) then one selects *γ*_1_(*t*) such that $b_{1}\leq h_{1}$ for *t* ∈ [1, 3], and *γ*_2_(*t*) such that $b_{2}\leq h_{2}$ for *t* ∈ [2, 4]; 4) finally, the composite barrier function is formed as $b=-\ln\left(\exp\left(-b_{1}\right)+\exp\left(-b_{2}\right)\right)$ .

**FIGURE 4:**
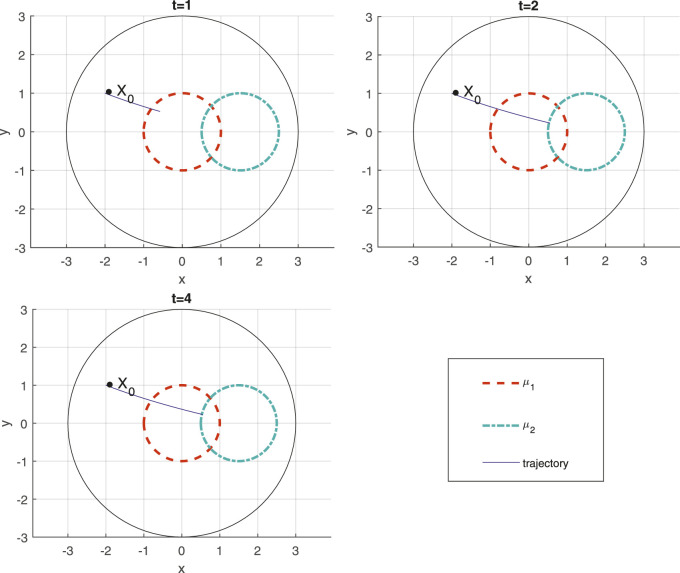
Trajectory of the robot for STL task *φ*_2_ (29) at different time instances, with time label at the top of each figure.

This process renders (29) not satisfiable. To see this, focus on the time interval *t* ∈ [2, 3] when the robot needs to visit and remain in the intersection of *μ*_1_ and *μ*_2_. Note that in this time interval, there must be *γ*_1_(*t*) ≤ 1 and *γ*_2_(*t*) ≤ 1 to ensure $b_{1}\leq h_{1}$ and $b_{2}\leq h_{2}$ , respectively. However, since the robot needs to be located somewhere in the intersection of *μ*_1_ and *μ*_2_, it must be *γ*_1_(*t*) ≥ 0.5 and *γ*_2_(*t*) ≥ 0.5 to ensure that $b_{1}\geq 0$ and $b_{2}\geq 0$ . As a result, for a any legitimate choice of *γ*_1_(*t*) and *γ*_2_(*t*), there will be $0\leq b_{1}\leq 0.5$ and $0\leq b_{2}\leq 0.5$ . This results in a composite barrier function b<0 in *t* ∈ [2, 3] for all legitimate choices of *γ*_1_(*t*) and *γ*_2_(*t*). While there exist solutions to satisfy the specification (keep each $b_{1}$ and $b_{2}$ non-negative), the conservatism introduced by the under-approximation of the minimum operator (corresponds to the conjunction in the STL formula) by the smooth exponential summation does not permit the satisfaction of (29). In contrast, the non-smooth formulation of this paper can handle (29) successfully. Figure 4 depicts snapshots of the robot’s path generated by the control design of Section 4.2, satisfying the STL task of (29). Having said that, it can be noted that there can be ways for this satisfiability gap to be reduced, as part of the QP problem—although, theoretically, it can never be completely eliminated.

### 5.3 Application to Robot-Child Interaction

**FIGURE 5:**
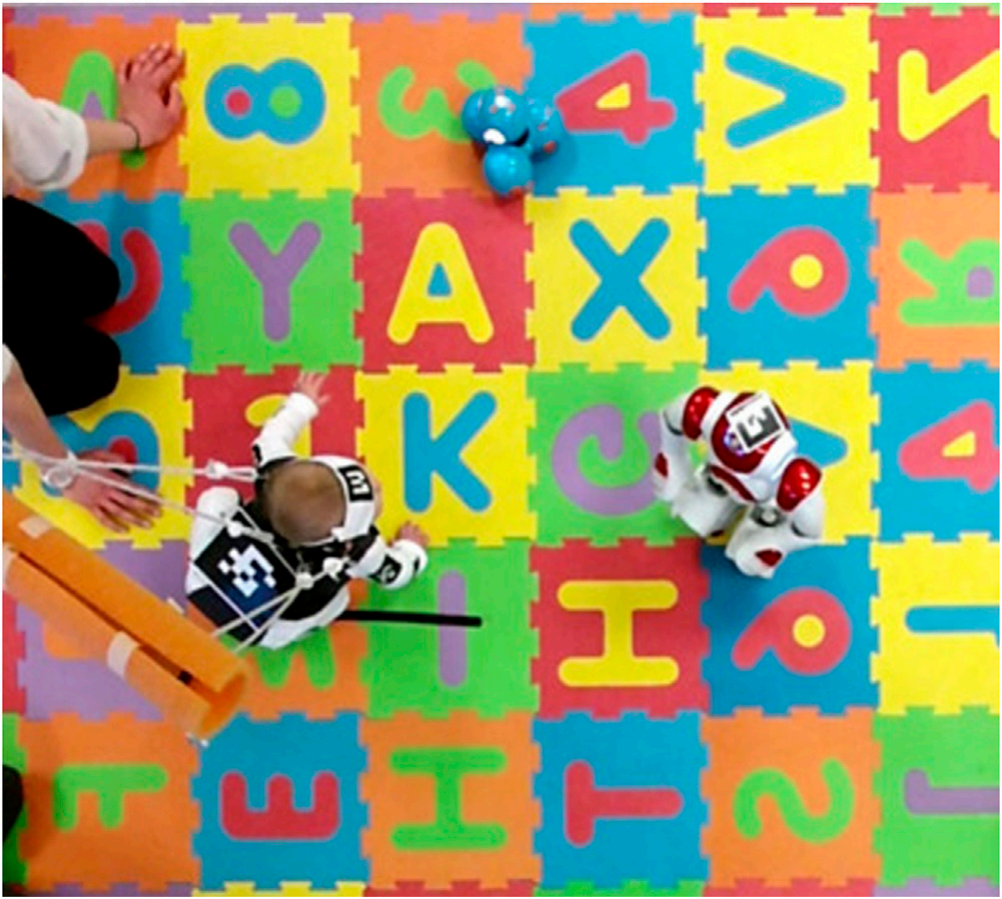
Instance of play-based child-robot social interaction. Two robots are visible in the scene: a small humanoid Nao, and a differential-drive small mobile robot toy Dash.

This section demonstrates how the nonsmooth CBF theory can be applied in the context of early pediatric motor rehabilitation, to regulate play-based social interaction between infants and mobile robots. The primary clinical objective of these mobile robots is to encourage infant mobility through interactive gameplay. Figure 5 shows an (enriched, in terms of stimuli) robot-assisted motor rehabilitation environment for infants involving two robots engaged in free-play activities with an infant.

The robots shown Figure 5 have been remotely controlled during the studies conducted, with the operators following a pre-determined look-up table of appropriate robot responses to infant reactions. Similar to other instances of Human Robot Interaction (HRI) application reported in literature (McGhan et al., 2015; Zehfroosh et al., 2017), a Markovian model is used to model the interaction at the high level. The parameters of this Markovian model are learned through observations in sessions with human subjects (Zehfroosh et al., 2017). Synchronized video from a network of surrounding cameras provided input to action recognition machine learning algorithms capable of identifying certain infant behaviors of interest, such as walking, crawling, standing, sitting, etc., as well as transitions between them (Kokkoni et al., 2020). In an envisioned fully automated version of this robot-assisted rehabilitation environment, robots could receive direct feedback regarding the child’s reactions and adapt their gameplay behavior accordingly in order to further encourage infant mobility.

In specific gameplay scenarios involved in the HRI protocol followed, infants and robots were playing a game of tag, in which the robot had a small set of options with regards to its play-based interaction with the child: close the distance to the infant; increase the distance to the infant; stand still (Kokkoni et al., 2020). Analysis of session data from a small number of subjects seemed to point to a new hypothesis according to which the type of robot behavior that usually triggers infant motor responses rarely involves single atomic actions, but is rather more complex involving several actions in temporal succession. For instance, it looked as if the robot could convey a social non-verbal cue such as “follow me” if it initially approached the child within about 1 m in distance, stood still for a short time interval, then attempted to increase the distance slightly, before repeating in a back-and-forth moving pattern. Motivated by these observations, we have subsequently conjectured that robot responses modeled in an LTL framework may be more effective in triggering the desired subject responses (Zehfroosh and Tanner, 2019). The STL framework described in this paper allows us to bring this HRI method to a new level, including timing constrains.

**FIGURE 6:**
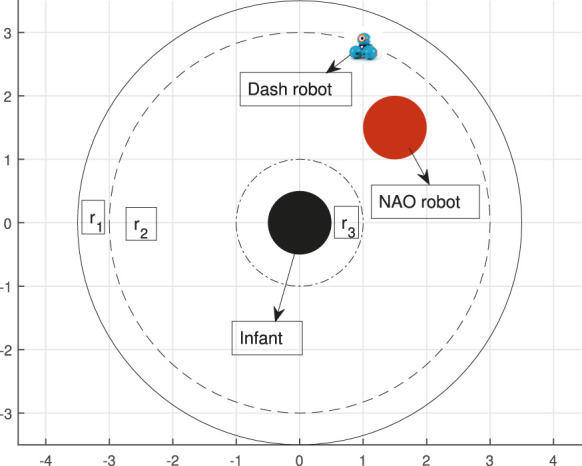
Schematic of Dash’s robot workspace.

To see how this could work in the context of the HRI scenario of Figure 5, consider a circular 2D robot workspace for Dash at the time when the “follow me” social cue is to be given (see Figure 6). Divide this workspace into three labeled regions *R* = {*r*_1_, *r*_2_, *r*_3_}, with the robot initialized in region *r*_2_. The desired back-and-forth moving pattern for the “follow me” task can be encoded by a random selection of some specific subregions of interest inside *r*_1_ and inside *r*_2_. Construct now an STL specification that requires the robot to visit those regions in order and with appropriate timing. For example, for three regions defined geometrically as in Table 3,the following STL behavior specification (in the form of formula *φ*_3_) for the robot can be defined as a way to signal “follow me” within 10 s to its human playmate:

$$\varphi_{3}=\left({□}_{\left[2,4\right]}\mu_{1}\right)\wedge \left({♢}_{\left[5,6\right]}\mu_{2}\right)\wedge \left({□}_{\left[8,10\right]}\mu_{3}\right)\;.$$ (30)

**FIGURE 7:**
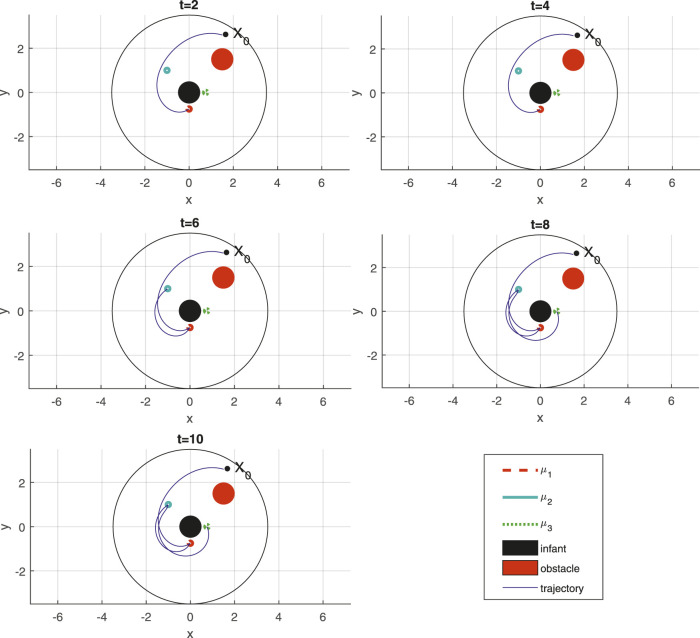
Trajectory of the Dash for STL task *φ*_3_ (30) at different time instances, with time label at the top of each figure.

Figure 7 presents Dash’s trajectory for STL formula *φ*_3_ given in (30), realized through the nonsmooth control barrier navigation function methodology of Section 4. Just like the motion planning scenario of Section 5.1, the robot path is shown in terms of snapshots at important time instances that attempt to illustrate the satisfaction of the STL specification (30).

**TABLE 3:** Geometric characteristics of regions of interest for a child-robot interaction scenario. *μ*_1_ and *μ*_3_ are inside *r*_1_ and *μ*_2_ is inside *r*_2_.

Predicate	Region center position	Radius
*μ*_1_	(0,−0.75)^⊺^	0.1
*μ*_2_	(−1,−1)^⊺^	0.1
*μ*_3_	(0.75,0)^⊺^	0.1

## 6 Conclusion

By now it is known that motion planning and control synthesis STL can be facilitated through the use of the concept of the control barrier function (CBF). This process obviates the need for model checking as a means of obtaining control laws that implement an STL specification, but still comes at the cost of utilizing a restricted fragment of STL and having to solve a QP problem in each cycle of the control loop. The incorporation of navigation functions as the base for the construction of CBFs, as advocated in this paper, is shown here to be advantageous because it alleviates the computational cost of utilizing CBFs in STL control synthesis. What is more, when the CBFs are combined through nonsmooth mappings as a means of encoding Boolean logic, the construction not only allows for covering larger class of STL specifications in comparison with the existing barrier-function STL planning methods, but also relaxes the conservativeness of existing smooth compositional formulations, and allows the resulting control laws to inherit some of the performance guarantees in terms of convergence and safety afforded by feedback motion plans based on navigation functions. Finally, the nonsmooth approach to combining navigation CBFs allows to expand the fragment of STL covered so that includes disjunctions at no apparent computational cost. The methodology described in this paper can prove useful in applications where robots are called to perform complex and temporally-dependent tasks. An example of such an application, which this paper highlights, is found in the context of pediatric robot-assisted motor rehabilitation.

## Data Availability

The original contributions presented in the study are included in the article/Supplementary Material, further inquiries can be directed to the corresponding author.

## Footnotes

1 A smooth version of such a function can be implemented based on the construction of Boothby (1986).

2 For this to happen, the two clauses of the STL formula corresponding to $b_{1}\left(x,t\right)$ and $b_{2}\left(x,t\right)$ will appear to be in conflict and each require that the system moves in exactly opposite directions; e.g., on the line *x* = 0 with, say, ${\text{b}}_{1}={\text{e}}^{-t}+\frac{{\left(x-a\right)}^{2}+y^{2}}{1+{\left(x-a\right)}^{2}+y^{2}}$ , and ${\text{b}}_{2}={\text{e}}^{-1}+\frac{{\left(x+a\right)}^{2}+y^{2}}{1+{\left(x+a\right)}^{2}+y^{2}}$ .

## References

[B1] Ames A. D. Coogan S. Egerstedt M. Notomista G. Sreenath K. Tabuada P. (2019). “Control Barrier Functions: Theory and Applications,” in 18th European Control Conference, Naples, Italy, 25-28 June 2019, 3420–3431. 10.23919/ECC.2019.8796030

[B2] Ames A. D. Xu X. Grizzle J. W. Tabuada P. (2016). Control Barrier Function Based Quadratic Programs for Safety Critical Systems. IEEE Trans. Automatic Control. 62, 3861–3876.

[B3] Bacciotti A. Ceragioli F. (2006). Nonpathological Lyapunov Functions and Discontinuous Carathéodory Systems. Automatica 42, 453–458. 10.1016/j.automatica.2005.10.014

[B4] Bacciotti A. Ceragioli F. (1999). Stability and Stabilization of Discontinuous Systems and Nonsmooth Lyapunov Functions. Esaim: Cocv 4, 361–376. 10.1051/cocv:1999113

[B5] Bacciotti A. Rosier L. (2005). Liapunov Functions and Stability in Control Theory. Berlin, Germany: Springer Science and Business Media.

[B6] Boothby W. M. (1986). An Introduction to Differentiable Manifolds and Riemannian Geometry. Cambridge, Massachusetts: Academic Press.

[B7] Campos J. J. Anderson D. I. Barbu-Roth M. A. Hubbard E. M. Hertenstein M. J. Witherington D. (2000). Travel Broadens the Mind. Infancy 1, 149–219. 10.1207/s15327078in0102_1 32680291

[B8] Chen C. Li C. Tanner H. G. (2020). Navigation Functions with Non-point Destinations and Moving Obstacles. Proc. IEEE Am. Control. Conf., 2532–2537. 10.23919/acc45564.2020.9147243

[B9] Clarke F. H. (1990). Optimization and Nonsmooth Analysis. Philadelphia, Pennsylvania: Society for Industrial and Applied Mathematics.

[B10] Clearfield M. W. (2004). The Role of Crawling and Walking Experience in Infant Spatial Memory. J. Exp. Child Psychol. 89, 214–241. 10.1016/j.jecp.2004.07.003 15501452

[B11] Glotfelter P. Buckley I. Egerstedt M. (2019). Hybrid Nonsmooth Barrier Functions with Applications to Provably Safe and Composable Collision Avoidance for Robotic Systems. IEEE Robot. Autom. Lett. 4, 1303–1310. 10.1109/lra.2019.2895125

[B12] Glotfelter P. Cortés J. Egerstedt M. (2017). Nonsmooth Barrier Functions with Applications to Multi-Robot Systems. IEEE Control. Syst. Lett. 1, 310–315. 10.1109/lcsys.2017.2710943

[B13] Göksun T. Hirsh-Pasek K. Michnick Golinkoff R. (2010). Trading Spaces: Carving up Events for Learning Language. Perspect. Psychol. Sci. 5, 33–42. 10.1177/1745691609356783 26162061

[B14] Gundana D. Kress-Gazit H. (2021). Event-based Signal Temporal Logic Synthesis for Single and Multi-Robot Tasks. IEEE Robot. Autom. Lett. 6, 3687–3694. 10.1109/lra.2021.3064220

[B15] Higgins C. I. Campos J. J. Kermoian R. (1996). Effect of Self-Produced Locomotion on Infant Postural Compensation to Optic Flow. Developmental Psychol. 32, 836–841. 10.1037/0012-1649.32.5.836

[B16] Jones A. M. Leahy K. Vasile C. Sadraddini S. Serlin Z. Tron R. (2019). “Scratchs: Scalable and Robust Algorithms for Task-Based Coordination from High-Level Specifications,” in International Symposium on Robotics Research (Piscataway, NJ: IEEE), 1–16. 10.1109/TRO.2021.3130794

[B17] Kokkoni E. Mavroudi E. Zehfroosh A. Galloway J. C. Vidal R. Heinz J. (2020). Gearing Smart Environments for Pediatric Motor Rehabilitation. J. Neuroeng Rehabil. 17, 16–15. 10.1186/s12984-020-0647-0 32041623 PMC7011606

[B18] Li C. Tanner H. G. (2018). Navigation Functions with Time-Varying Destination Manifolds in Star-worlds. IEEE Trans. Robot 35, 35–48. 10.1109/TRO.2018.2875421 30853871 PMC6402493

[B19] Lindemann L. Dimarogonas D. V. (2020). Barrier Function Based Collaborative Control of Multiple Robots under Signal Temporal Logic Tasks. IEEE Trans. Control. Netw. Syst. 7, 1916–1928. 10.1109/tcns.2020.3014602

[B20] Lindemann L. Dimarogonas D. V. (2019a). Control Barrier Functions for Multi-Agent Systems under Conflicting Local Signal Temporal Logic Tasks. IEEE Control. Syst. Lett. 3, 757–762. 10.1109/lcsys.2019.2917975

[B21] Lindemann L. Dimarogonas D. V. (2018). Control Barrier Functions for Signal Temporal Logic Tasks. IEEE Control Syst. Lett. 3, 96–101. 10.1109/LCSYS.2018.2853182

[B22] Lindemann L. Dimarogonas D. V. (2019b). Feedback Control Strategies for Multi-Agent Systems under a Fragment of Signal Temporal Logic Tasks. Automatica 106, 284–293. 10.1016/j.automatica.2019.05.013

[B23] Lindemann L. Dimarogonas D. V. (2021). Funnel Control for Fully Actuated Systems under a Fragment of Signal Temporal Logic Specifications. Nonlinear Anal. Hybrid Syst. 39, 100973. 10.1016/j.nahs.2020.100973

[B24] Lindemann L. Verginis C. K. Dimarogonas D. V. (2017). “Prescribed Performance Control for Signal Temporal Logic Specifications,” in 2017 IEEE 56th Annual Conference on Decision and Control (CDC), Melbourne, VIC, Australia, 12-15 Dec. 2017 (IEEE), 2997–3002. 10.1109/cdc.2017.8264095

[B25] Liu Z. Dai J. Wu B. Lin H. (2017). Communication-aware Motion Planning for Multi-Agent Systems from Signal Temporal Logic Specifications. IEEE Am. Control. Conf., 2516–2521. 10.23919/acc.2017.7963331

[B26] Maler O. Nickovic D. (2004). “Monitoring Temporal Properties of Continuous Signals,” in Formal Techniques, Modelling and Analysis of Timed and Fault-Tolerant Systems (Berlin, Heidelberg: Springer), 152–166. 10.1007/978-3-540-30206-3_12

[B27] McGhan C. L. R. Nasir A. Atkins E. M. (2015). Human Intent Prediction Using Markov Decision Processes. J. Aerospace Inf. Syst. 12, 393–397. 10.2514/1.i010090

[B28] Raman V. Donzé A. Maasoumy M. Murray R. M. Sangiovanni-Vincentelli A. Seshia S. A. (2014). “Model Predictive Control with Signal Temporal Logic Specifications,” in 53rd IEEE Conference on Decision and Control, Los Angeles, CA, USA, 15-17 Dec. 2014, 81–87. 10.1109/CDC.2014.7039363

[B29] Rimon E. Koditschek D. E. (1992). Exact Robot Navigation Using Artificial Potential Functions. IEEE Trans. Robot. Automat. 8, 501–518. 10.1109/70.163777

[B30] Sadraddini S. Belta C. (2015). “Robust Temporal Logic Model Predictive Control,” in 53rd IEEE Annual Allerton Conference on Communication, Control, and Computing, 29 Sept.-2 Oct. 2015Monticello, IL, USA, 772–779. 10.1109/ALLERTON.2015.7447084

[B31] Sun J. Tanner H. G. (2015). Constrained Decision-Making for Low-Count Radiation Detection by mobile Sensors. Auton. Robot 39, 519–536. 10.1007/s10514-015-9468-6

[B32] Zehfroosh A. Kokkoni E. Tanner H. G. Heinz J. (2017). “Learning Models of Human-Robot Interaction from Small Data,” in 25th Mediterranean Conference on Control and Automation, Valletta, Malta, 3-6 July 2017, 223–228. 10.1109/MED.2017.7984122 PMC5826574 29492408

[B33] Zehfroosh A. Tanner H. G. (2019). “Reactive Motion Planning for Temporal Logic Tasks without Workspace Discretization,” in IEEE American Control Conference, Philadelphia, PA, USA, 10-12 July 2019, 4872–4877. 10.23919/ACC.2019.8814420

